# Urate crystal deposition and bone erosion in gout: ‘inside-out’ or ‘outside-in’? A dual-energy computed tomography study

**DOI:** 10.1186/s13075-016-1105-z

**Published:** 2016-09-15

**Authors:** Patapong Towiwat, Anthony J. Doyle, Gregory D. Gamble, Paul Tan, Opetaia Aati, Anne Horne, Lisa K. Stamp, Nicola Dalbeth

**Affiliations:** 1 Department of Medicine, Faculty of Medical and Health Sciences, University of Auckland, 85 Park Road, Grafton, Auckland New Zealand; 2 Department of Medicine, Naresuan University, Phitsanulok, Thailand; 3 Department of Anatomy and Medical Imaging, University of Auckland, Auckland, New Zealand; 4 Department of Medicine, University of Otago, Christchurch, New Zealand

**Keywords:** Gout, Tophus, Urate, Bone, Erosion, Dual-energy computed tomography

## Abstract

**Background:**

It is currently unknown whether bone erosion in gout occurs through an ‘inside-out’ mechanism due to direct intra-osseous crystal deposition or through an ‘outside-in’ mechanism from the surface of bone. The aim of this study was to examine the mechanism (‘outside-in’ vs. ‘inside-out’) of monosodium urate (MSU) crystal deposition in bone erosion in gout. Specifically, we used three-dimensional dual-energy computed tomography (DECT) to analyse the positional relationship between bone and MSU crystal deposition in tophaceous gout, and to determine whether intra-osseous crystal deposition occurs in the absence of erosion.

**Methods:**

One hundred forty-four participants with gout and at least one palpable tophus had a DECT scan of both feet. Two readers independently scored all metatarsal heads (1433 bones available for scoring). For bones in contact with urate, the bone was scored for whether urate was present within an erosion, on the surface of bone or within bone only (true intra-osseous deposit). Data were analysed using generalised estimating equations.

**Results:**

Urate in contact with bone was present in 370 (54.3 %) of 681 joints with urate deposition. For those bones in contact with urate, deposition was present on the surface of bone in 143 (38.6 %) of 370 joints and within erosion in 227 (61.4 %) of 370. True intra-osseous urate deposition was not observed at any site (*p* < 0.0001). For all bones with apparent intra-osseous deposition in one plane, examination in other planes revealed urate deposition within an *en face* erosion.

**Conclusions:**

In tophaceous gout, MSU crystal deposition is present within the joint, on the bone surface and within bone erosion, but it is not observed within bone in the absence of a cortical break. These data support the concept that MSU crystals deposit outside bone and contribute to bone erosion through an ‘outside-in’ mechanism.

## Background

Bone erosion is a frequent consequence of tophaceous gout. Advanced imaging techniques such as conventional computed tomography and magnetic resonance imaging have demonstrated a close relationship between tophus and bone erosion [1, 2]. The tophus is an organized structure consisting of monosodium urate (MSU) crystals and chronic inflammatory tissue [3]. Dual-energy computed tomography (DECT) is a recently developed imaging method that allows non-invasive detection of urate [4]. DECT studies have also shown a close relationship between bone erosion and MSU crystal deposition in tophaceous gout [5].

Bone erosion occurs in other forms of inflammatory arthritis, including rheumatoid arthritis (RA). Histological and imaging studies have implicated both invasion of inflamed synovial tissue or pannus into bone (‘outside-in’) and cytokine-mediated osteitis visualized as bone marrow oedema (‘inside-out’) as potential mechanisms for bone erosion in RA [6–8].

Some investigators have postulated that MSU crystals can form within the Haversian canals, leading to true intra-osseous deposition [9]. However, in joints affected by gout, MSU crystals are observed primarily within synovial fluid, synovium, articular cartilage, and bone in the subchondral region [10]. Histological analysis of joints affected by gout show that MSU crystals are frequently deposited at the surface of cartilage [10]. Similarly, in imaging studies using high-resolution ultrasonography, the double-contour sign is frequently observed; this sign is thought to represent MSU crystals coating the articular cartilage [11]. Collectively, pathology and imaging observations support the concept that bone erosion in gout occurs through an ‘outside-in’ mechanism whereby MSU crystals deposit on the surface of articular cartilage surface or within synovium and then interact with bone cells to develop erosion. If this is the case, we would anticipate that MSU crystals would be present both on the surface of bone and within bone erosion, but that intra-osseous MSU crystal deposition without a cortical break (erosion) would not occur.

The aim of this study was to examine the mechanism (‘outside-in’ vs. ‘inside-out’) of MSU crystal deposition in bone erosion in gout. Specifically, we used three-dimensional DECT imaging to analyse the positional relationship between bone and MSU crystal deposition in tophaceous gout, and to determine whether intra-osseous crystal deposition occurs in the absence of erosion.

## Methods

One hundred forty-four people with tophaceous gout were prospectively recruited from rheumatology clinics in Auckland, New Zealand. All participants had gout according to the 1977 American Rheumatism Association classification criteria [12] and at least one palpable tophus found in a clinical examination. The New Zealand Ministry of Health Ethics Committee approved this study. All participants provided written informed consent before inclusion in the study.

DECT scans of the feet were performed using a dual-x-ray tube 128 detector row scanner (SOMATOM Definition Flash; Siemens Medical, Erlangen, Germany). The patients were positioned feet first in a supine position with the feet in a plantar flexion position. The scan was acquired in a craniocaudal direction, starting proximally 5 cm from the ankle joint to the toe tips. Both ankles and feet were scanned axially in one helical acquisition. All scans were taken with the same image protocol: acquisition at 128 × 0.6 mm and pitch of 0.7. X-ray tube 1 was operated at 80 kV/260 mA and tube 2 at 140 kV/130 mA. The images were reconstructed on a bone algorithm, 512 × 512 matrix, into 0.75-mm slices with a 0.5-mm increment. Additional reconstructions were done on a soft tissue algorithm, 512 × 512 matrix, also into a 0.75-mm slice with a 0.5-mm increment. The images were viewed as 0.75-mm slices using a picture archiving and communication system. A proprietary workstation (syngo MultiModality Workplace; Siemens Medical) was used with proprietary software (syngo MMWP VE 36A 2009; Siemens Medical). For the 80-kV images, fluid was set at 50 Hounsfield units (HU), the ratio for urate at 1.36, minimum HU at 150 and smoothing range at 4. For the 140-kV images, fluid was set at 50 HU and maximum HU at 500.

Two trained readers (PTo and ND) independently scored all metatarsal heads (1433 bones available for scoring). Each site was initially scored for the presence or absence of urate deposition within the joint. If urate was present within the joint, the presence of urate in contact with bone was then scored. For bones in contact with urate, the site was then scored for whether urate was present within an erosion (a sharply demarcated area of focal bone loss seen in two planes, with a cortical break in at least one plane), and if not within an erosion, on the surface of bone or within bone only (as a true intra-osseous deposit). In the case of reader disagreement, agreement was reached in a consensus re-scoring exercise. Pre-consensus inter-reader κ values for all scoring were >0.93.

Data were analysed using SAS version 9.4 software (SAS Institute Inc., Cary, NC, USA). Means with SDs and percentages were used to describe the clinical characteristics of participants. Differences between the location of urate deposits in each and all metatarsal heads were modelled taking into account clustering of results within individuals using generalised estimating equations and the GENMOD procedure of SAS. Confidence intervals for proportions were calculated by using Fisher’s exact test with the mid-P method using www.OpenEpi.com [13]. All tests were two-tailed, and *p* values less than 0.05 were considered statistically significant.

## Results

### Participants

Participant characteristics are shown in Table 1. Participants were predominantly men (93.1 %). The mean (SD) age of the sample was 59 years, and median disease duration was 22 years. More than half of the participants were of Māori or Pacific ethnicity. Most (97.3 %) were receiving urate-lowering therapy. The mean (SD) serum urate at the time of scanning was 0.40 (0.12) mmol/L. There were four participants who were not receiving urate-lowering therapy due to patient preference or intolerance to urate-lowering therapies. These participants had numerically fewer palpable tophi than the group overall (median number of tophi 2).

**Table 1:** Clinical features of study participants

Male sex, *n* (%)	134 (93.1 %)
Age, mean (SD)	59 (12) years
Ethnicity, *n* (%)	
Māori Pacific Non-Māori, non-Pacific	16 (11.0 %) 63 (43.8 %) 65 (45.1 %)
Disease duration, mean (SD)	22 (12) years
Number of tophi, median (range)	4.5 (1–41)
Urate-lowering therapy use, *n* (%)	
None Allopurinol Probenecid Benzbromarone Combination of allopurinol plus uricosuric	4 (2.7 %) 126 (87.5 %) 4 (2.7 %) 3 (2.1 %) 7 (4.9 %)
Serum urate, mean (SD)	0.40 (0.12) mmol/L

### Positional relationship between bone and MSU crystal deposition

**Fig. 1:**
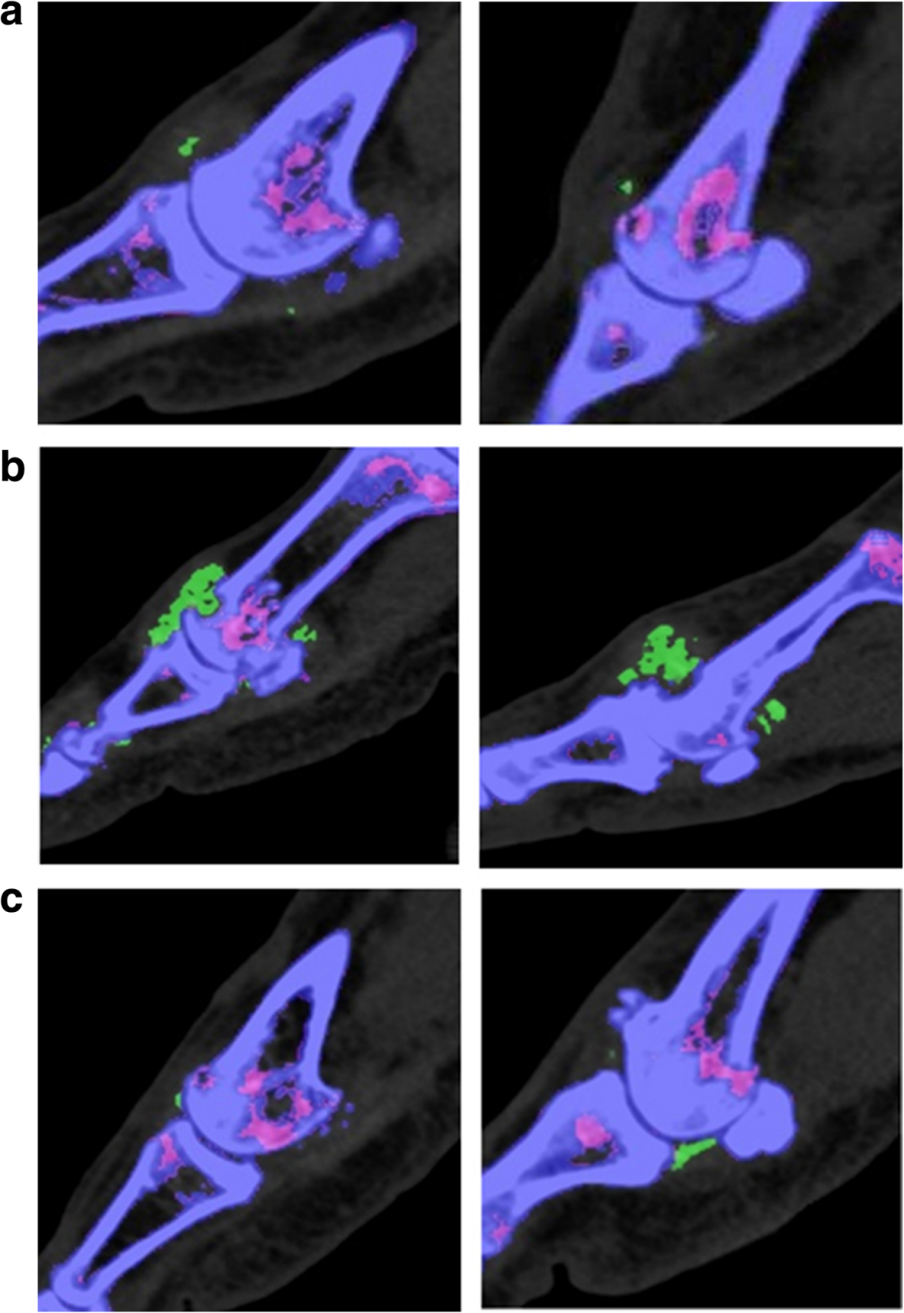
Sagittal images of the metatarsal heads from different patients. Images show examples of (**a**) urate deposition inside the joint but not in contact with bone, (**b**) urate deposition in contact with bone and within an erosion, and (**c**) urate deposition in contact with bone and on the surface of bone. Urate is colour-coded as *green* and bone as *purple*

For all metatarsal heads, urate deposition in the joint was present in 681 (47.5 %) of 1443 sites (Fig. 1a). Urate in contact with bone was present in 370 (54.3 %) of 681 joints with urate deposition. For those bones in contact with urate, deposition was present within erosion in 227 (61.4 %) of 370 joints (Fig. 1b) and on the surface of bone in 143 (38.6 %) of 370 (Fig. 1c).

**Fig. 2:**
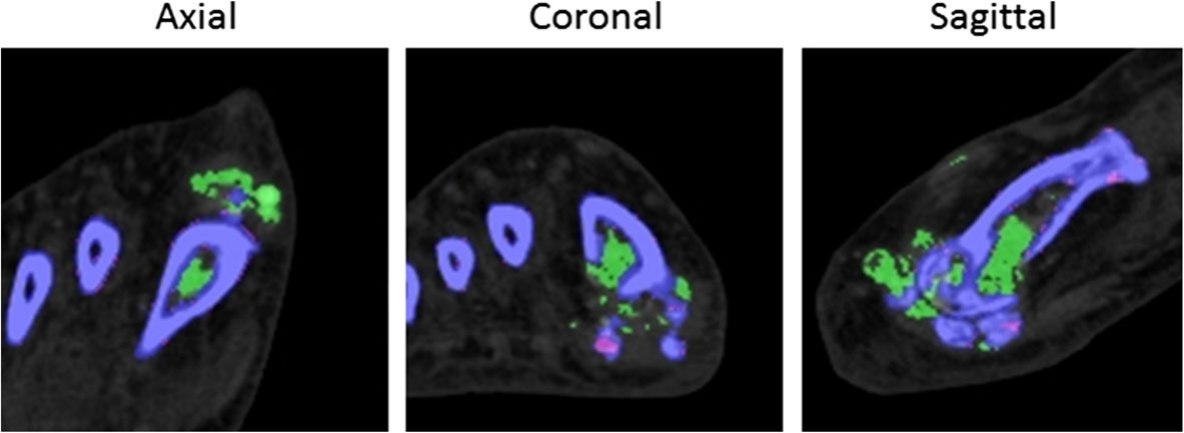
Example of apparent metatarsal intra-osseous urate deposit in the axial plane, with images in other planes showing urate within an erosion. Urate is colour-coded as *green* and bone as *purple*

True intra-osseous urate deposition was not observed at any site (compared with deposition in erosions and on the surface of bone; *p* < 0.0001). For all bones with apparent intra-osseous deposition in one plane, examination in other planes demonstrated urate deposition within an *en face* erosion (Fig. 2).

Table 2 shows the distribution of urate deposition at each metatarsal head and at all bone sites. Urate deposition was present most frequently at the first metatarsal head. Similarly, urate deposition in contact with bone and at the site of erosion was also most often observed at the first metatarsal head.

**Table 2:** The distribution of urate deposits in the metatarsal heads (*n* = 144 patients)

	Metatarsal head 1 (*n* = 288)	Metatarsal head 2 (*n* = 287)	Metatarsal head 3 (*n* = 287)	Metatarsal head 4 (*n* = 287)	Metatarsal head 5 (*n* = 284)	All metatarsal heads (*n* = 1433)
Urate deposit in joint, *n* (%) (95 % CI)	230 of 288 (79.9 %) (74.9–84.1 %)	139 of 287 (48.4 %) (42.7–54.2 %)	101 of 287 (35.2 %) (29.9–40.9 %)	85 of 287 (29.6 %) (24.6–35.1 %)	126 of 284 (44.4 %) (38.7–50.2 %)	681 of 1433 (47.5 %) (45.0–50.1 %)
Urate deposit in contact with bone, *n* (%) (95 % CI)	169 of 230 (73.5 %) (67.4–78.8 %)	64 of 139 (46.0 %) (38.0–54.3 %)	38 of 101 (37.6 %) (28.8–47.4 %)	27 of 85 (32 %) (23–42 %)	72 of 126 (57.1 %) (48.4–65.5 %)	370 of 681 (54.3 %) (50.6–58.0 %)
Urate deposit on bone surface only, *n* (%) (95 % CI)	40 of 169 (23.7 %) (17.9–30.6 %)	39 of 64 (61 %) (49–72 %)	27 of 38 (71 %) (52–83 %)	14 of 27 (52 %) (34–69 %)	23 of 72 (32 %) (22–43 %)	143 of 370 (38.6 %) (33.8–43.7 %)^a^
Urate deposit at site of erosion, *n* (%) (95 % CI)	129 of 169 (76.3 %) (69.4–82.1 %)	25 of 64 (39 %) (28–51 %)	11 of 38 (28.9 %) (17–5 %)	13 of 27 (48.1 %) (31–66 %)	49 of 72 (68.1 %) (57–78 %)	227 of 370 (61.4 %) (56.3–66.2 %)^a^
True intra-osseous urate deposit, *n* (%) (95 % CI)	0 of 169 (0 %) (0–2 %)	0 of 64 (0 %) (0–6 %)	0 of 38 (0 %) (0–9 %)	0 of 27 (0 %) (0–13 %)	0 of 72 (0 %) (0–4 %)	0 of 370 (0 %) (0–1 %)^a^

^a^ *p* < 0.0001

## Discussion

This study demonstrates that MSU crystal deposition in tophaceous gout is present within the joint, on the bone surface and within bone erosion, but is not observed within bone in the absence of a cortical break. In prior pathological studies of gout, researchers have reported MSU crystal deposition on the surface of bone, within erosion and in intra-osseous locations, and some investigators have postulated that MSU crystals can form directly within the Haversian canals of bone [14]. However, this question has been difficult to address using standard pathological methods, as analysis of microscopic slides may not allow assessment of the entire bone surface. The ability of DECT to reveal both urate deposition and the entire bone in three dimensions has provided new insight into this question. Although our analysis does not absolutely exclude the possibility that MSU crystals can form within bone, our DECT data strongly support the concept that MSU crystals form deposits outside bone prior to development of erosion.

The cellular mechanisms of bone erosion can be considered in the context of these imaging results. Laboratory studies have demonstrated that MSU crystals can interact with bone and joint cells both directly and indirectly to promote bone erosion. Numerous osteoclasts are present at the interface between bone and tophus in erosive gout, and MSU crystals promote osteoclastogenesis through interactions with stromal cells [15]. MSU crystal-induced production of catabolic enzymes and cytokines that promote osteoclastogenesis by synovial fibroblasts, macrophages and chondrocytes may also contribute to bone erosion [16–19]. MSU crystals also directly induce chondrocyte cell death, which may compromise integrity of cartilage and allow focal contact of MSU crystals with sub-chondral bone [17].

This analysis has some limitations. We focused only on the metatarsal heads, and it is possible that different mechanisms of erosion might occur at other bone sites. The sites for scoring were selected for analysis on the basis of high frequency of disease, particularly in the first metatarsal head. Initial inter-reader reliability testing also demonstrated that these sites were associated with the most reproducible scores (data not shown). Consistent with the epidemiology of gout, most of the participants were men, and these findings may not be generalizable to women. DECT does have limitations of detection, and very small deposits of crystals may not be detected using this method [20]. Almost all participants were receiving urate-lowering therapy, with a wide range of serum urate concentrations at the time of scanning. It is possible that some patients may have had previous remodelling of urate deposits or bone erosion following urate-lowering therapy. To address this issue, the study was designed to include only sites with urate deposits evident on DECT scans in the bone analysis. We also acknowledge that the cross-sectional study design did not allow us to track the progression of urate deposition and erosion over time. For this reason, this analysis cannot conclusively prove that true intra-osseous deposition does not occur as a precursor to erosion. Nevertheless, the lack of any true intra-osseous deposition in this study of a large number of joints argues against this possibility.

## Conclusions

This systematic DECT analysis examining the three-dimensional positional relationship between bone and urate shows that MSU crystals are deposited on the bone surface and within bone erosion, but not as true intra-osseous depositions. These data support the concept that MSU crystals deposit outside bone and then contribute to bone erosion through an ‘outside-in’ mechanism.

## Abbreviations

DECT: Dual-energy computed tomography
HU: Hounsfield units
MSU: Monosodium urate
RA: Rheumatoid arthritis
